# Establishment and Characterization of a New Triple Negative Breast Cancer Cell Line from an Iranian Breast Cancer Tissue

**DOI:** 10.31557/APJCP.2019.20.6.1683

**Published:** 2019

**Authors:** Farzaneh Ghaderi, Fereshteh Mehdipour, Ahmad Hosseini, Abdolrasoul Talei, Abbas Ghaderi

**Affiliations:** 1 *Department of Immunology, *; 2 *Shiraz Institute for Cancer Research, *; 3 *Department of Surgery, School of Medicine, Shiraz University of Medical Sciences, Shiraz, Iran. *

**Keywords:** Breast cancer, cell line establishment, Triple-negative-, Pari-ICR

## Abstract

Breast cancer is the most common malignancy and the leading cause of cancer-related death among women worldwide. The underlying mechanisms for breast cancer development, especially in young women, are not completely understood. Although there are several experimental models to understand the biology of breast cancer such as immortalized cell lines, many of these cell lines have been in culture for decades and most of them have been derived from Caucasians or African-Americans. So, it is required to establish a new cell line derived from primary tumors and Asian women. In this study Pari-Institute for Cancer Research (Pari-ICR) was derived from the primary breast tumor of a 36-years old patient with invasive ductal carcinoma. We characterized the cell line by examining morphology, expression of different markers, and functional profile. Immunocytochemistry showed that this cell line does not express estrogen and progesterone receptors as well as human epidermal growth factor receptor 2 (HER2). Pari-ICR cell line expresses high levels of Vimentin, Ezrin, and S100 but does not express EpCAM, Cytokeratin19, Pan-cytokeratin, Nestin, and Desmin. Its doubling time of Pari-ICR was about 22h and was able to grow as colonies in soft agar. It displayed a higher ability of migration and invasion in comparison with MCF-7 cell line. This breast cancer cell line can serve as a model for understanding the molecular mechanisms of breast carcinogenesis. Moreover, it can be used as an appropriate resource to find novel biomarkers or assess new drugs.

## Introduction

Breast cancer is the most common malignancy and the leading cause of cancer-related death among women worldwide (Banin Hirata et al., 2014; Zhang et al., 2015). In Iran, breast cancer accounts for 24.6% of total cancers with the crude incidence rate of about 22.6 per 100,000 females, which even is increasing annually. According to Iran National Cancer Registry (NCR), it affects the Iranian women at least 10 years earlier compared to women in developed countries and about 21% of women with breast cancer are under the age of 40 years (Jazayeri et al., 2015; Otaghvar et al., 2015). Among different subtypes, triple-negative and HER-2 positive breast cancers are more prevalent in young women and tend to have more aggressive behaviors (Anders et al., 2009; Carey et al., 2010; Assi et al., 2013). 

Triple-negative breast cancers (TNBCs) are a heterogeneous group of tumors, both clinically and pathologically. These cancers are defined by the lack of express estrogen and progesterone receptors as well as human epidermal growth factor receptor 2 (HER2) expressions and account for approximately 15% of all breast cancers. TNBCs, which are significantly more frequent in women younger than 40 years, have a poor prognosis and reduced overall survival (Dent et al., 2007; Rapoport et al., 2014; Collignon et al., 2016).

Despite massive studies, the molecular pathways involved in breast cancer development, especially in young women, are not completely understood. Different experimental approaches can be applied to understand the biology of breast cancer such as those using immortalized cell lines. While cancer cell lines maintain many features of the tumor tissue (Neve et al., 2006; Vargo-Gogola and Rosen, 2007; Van Staveren et al., 2009), there are some difficulties with using them. For instance, the majority of them have been isolated from a metastatic tumor of older patients and many of these cell lines have been in culture for decades(Ku et al., 2013; Pandrangi et al., 2014). In addition, the genetic diversity of tumor cells may cause unpredictable tumor behavior and treatment failure. It has been recently evidenced that there is variability in the pharmacokinetics, pharmacodynamics, and tolerance of anticancer drugs among individuals with different ethnicities. In other words, ethnic variations have been found in anticancer drug response and in toxicity from anticancer drugs, especially among Asian and Caucasian cancer patients (Phan et al., 2009). Therefore, new cell lines derived from primary breast tumors of Asian young women are highly needed. The aim of this study is to describe the establishment and partial characterization of a triple negative breast cancer cell line derived from a primary tumor of 36-years old women with invasive ductal carcinoma of the breast.

## Materials and Methods

*Clinical history*

The cancer cell line was obtained from the primary breast tumor of a 36 years old patient with grade II invasive ductal carcinoma. This study was approved by the Ethics Committee of Shiraz University of Medical Sciences and an informed consent was obtained from patient.

The patient underwent a right-side mastectomy and axillary lymph node dissection. Metastases were found in 8 out of 16 excised lymph nodes. Under sterile condition, the tumor specimen was washed twice with phosphate-buffered saline (PBS) and minced with a scalpel into small pieces of approximately 2 mm^2^ in size. These small tissue pieces were seeded into a Petri dish, cultured in a complete DMEM/F12 medium (Gibco, USA), supplemented with 10% fetal bovine serum (FBS, Gibco, USA) and 1% penicillin-streptomycin (Bioidea, UK). After about 10 days, epithelial and mesenchymal cells were projected out of small tissue fragments. Next, the cells were enzymatically harvested (Trypsin 0.25%) and passaged with a 1:5 split ratio at the confluence of 80-90%. After 11 passages, tumor cells outcompeted the mesenchymal cells due to their faster growth and shorter doubling time. Pari-ICR were frozen periodically and stored in liquid nitrogen at various passages.

*Calculation of growth rate*

To calculate cell growth rate, the Pari-ICR cell line was plated at a concentration of 2.5× 10^4^in a DMEM/F12 medium supplemented with 10% FBS and 1% penicillin-streptomycin. Triplicate wells were counted after staining with trypan blue dye at 24-h intervals. The growth curve was plotted and doubling time calculated using free online doubling time calculating software (http://www.doubling-time.com).

*Colony-forming assay*

A two-layer method was performed. After trypsinization, a single cell suspension of the established cell line from 100 to 1,000 cells per plate were prepared in a solution containing 0.36% agar in DMEM/F12 supplemented with 10% FBS and 1% penicillin-streptomycin. The mixture was deposited on base layers (containing 0.75% agar with DMEM/F12 plus 10% FBS and 1% penicillin-streptomycin) and incubated at 37^o^C under 5% CO_2_. After 10 days, the numbers of colonies containing more than 50 cells were counted by an inverted microscope.

*Migration and invasion assays*

Migration and invasion assays were performed using transwell permeable inserts with or without coated Matrigel. After 24h of serum starvation, 5 × 10^4^ cells in 100µl serum-free DMEM/F12 with 1% penicillin-streptomycin were added to the upper chamber of the transwell, while the lower chamber of the transwell was filled with 600 μl DMEM supplemented with 10% FBS. After 24h of incubation at 37^o^C with 5% CO_2_, non-migratory cells on the upper surface of the membrane were removed with cotton swab. The migrated or invaded cells on the lower surface were fixed with ice-cold alcohol, stained with a solution containing 0.5% crystal violet in 10% ethanol, and counted under phase contrast microscope. The assay was performed in triplicate.

*Chromosomal Analysis*

To identify chromosomal abnormalities, karyotyping of Pari-ICR cell line was performed. Briefly, confluent cells were exposed to 0.2pg/ml Colcemid at 37^o^C for 2h and then detached by trypsinization. The harvested cells were treated with 0.5% potassium chloride solution for 30 min. After fixation with a fixative solution (methanol/glacial acetic acid 3:1), the cell suspension was spread on wet slides. Finally, the slides were colored in Gimsa staining solution for chromosome analysis.

*Comparative genomic hybridization (CGH)*

DNA amplification was performed using Sure PlexDNA amplification system according to the manufacturer’s protocol (Illumina, UK). After DNA amplification, control DNA [DNA from a normal male] and sample DNA were directly labeled with Cy3 and Cy5 Fluorophores, respectively. Sample DNA was hybridized overnight with a control DNA undercover slide. Finally, the array field was scanned by laser (Innopsys, France) and the microarray data on chromosome Loss/gain across 24 chromosomes were analyzed using Blue Fuse software (BlueGenome, UK).

*Immunofluorescent assay*

Pari-ICR cell line was seeded on coverslips at a density of 100 × 103 cells and grown for 24 h. The cells were then permeabilized with cold methanol for 15 min at room temperature. Blocking was done with 1% BSA (Biosera) in PBS containing 0.5% Tween 20 (PBST). Next, the slides were incubated overnight at 4^o^C with primary antibodies against Vimentin, Nestin, Ezrin, S100, Desmin, Pan-cytokeratin, and Cytokeratin19 [all from Santa Cruz]. After incubation, the cells were washed 3 times with PBST and incubated with corresponding secondary fluorescent antibodies (Goat anti-rabbit IgG- conjugated with FITC or goat anti-mouse IgG-conjugated with FITC, Santa Cruz) for 2h at room temperature. After washing with PBST, coverslips were mounted using amounting medium containing DAPI or PI and examined with a fluorescent microscope.

*Flow cytometry*

Expressions of cell surface markers were analyzed by flow cytometry. Cells were detached and washed with PBS twice, dissociated into single cell suspension, and incubated with fluorescent-conjugated monoclonal antibodies including anti CD73, CD90, CD166, CD29, CD24, CD44, CD10, CD45, CD34, CD105, CD133, and CD146(all from BD Bioscience, USA). After 30 min of incubation in the dark at 4^o^C, the cells were washed and resuspended in PBS and analyzed with FACS Calibur flow cytometer. Flow data were analyzed using FlowJo software version 7.6.2 (Tritar Inc., USA).

## Results

*Establishment of human breast cancer cell line*

Pari-ICR was established from a 36-year-old female patient, diagnosed with invasive ductal carcinoma of the breast.

**Figure 1:**
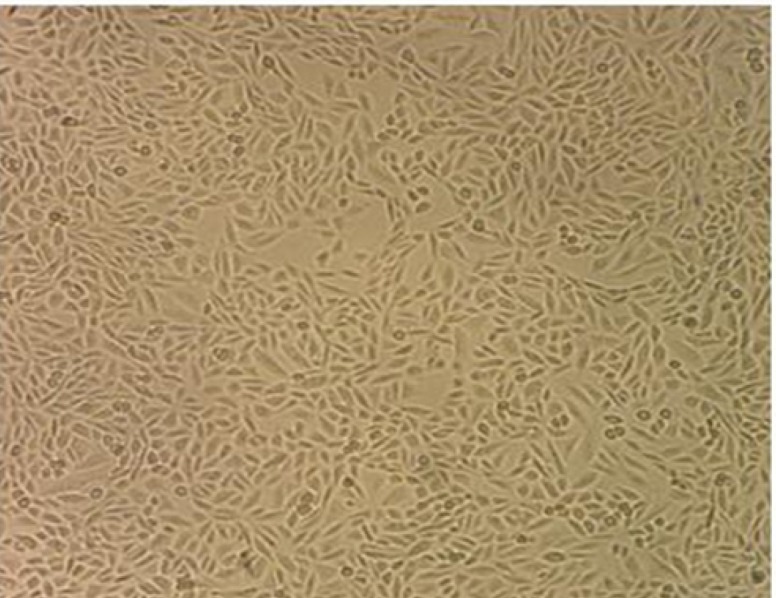
Cellular Morphology of Pari-ICR Cell Line in Cell Culture. The epithelial-like cells grew as a monolayer

Culture dish was examined daily using a phase contrast microscope. An outgrowth of tumor-like cells with a spindle shape was seen from breast explants 10 days after culture initiation. After 11 passages, the epithelial cells were enriched and a homogenous population was seen. These spindle-shaped cells grow as an adherent monolayer. So far, Pari-ICR cell line has been grown for 150 passages (Figure 1).

**Figure 2:**
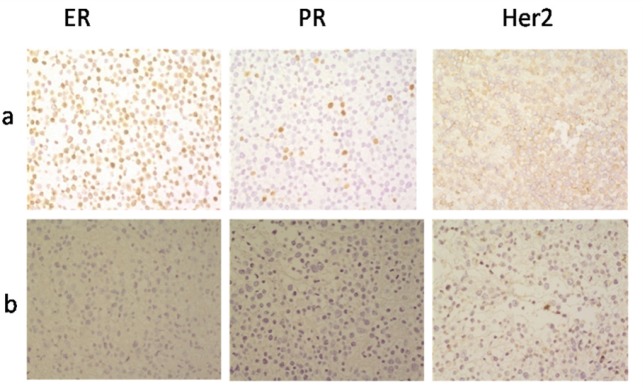
Immunocytochemical Profiles of Estrogen Receptor (ER), Progesterone Receptor (PR) and HER2 Receptor. (a) MCF-7 cell line: positive for ER (90%) and PR (10%) receptors, weakly positive for HER2/neu receptor. (b) Pari-ICR cell line: Negative for ER, PR and Her2/neureseptors. Images were taken at 400× magnification

Immunocytotochemical study revealed that Pari-ICR cell line was negative for ER, PR and HER2, while in the control cell line (MCF-7), ER and PR were positive in about 90% and 10% of the nucleus, respectively. HER2/neu had faint and incomplete membranous staining on MCF-7 (score 1+) (Figure 2).

*Characterization of the cell line*

*Growth characteristic and anchorage-independent growth in soft-agar*

**Figure 3:**
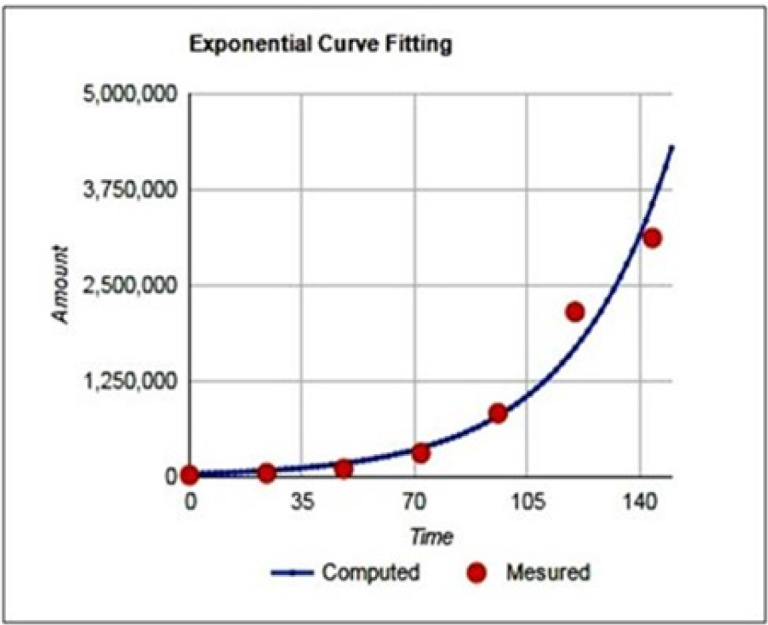
Growth Curve of Pari-ICR. Doubling time calculated using free online doubling time calculating software (http://www.doubling-time.com). The doubling time of Pari-ICR was about 22h

Doubling time of Pari-ICR was calculated at passages 50 during the exponential growth phase of the cell line by counting the number of cells for 6 days. The doubling time of Pari-ICR was about 22h (Figure 3).

**Figure 4:**
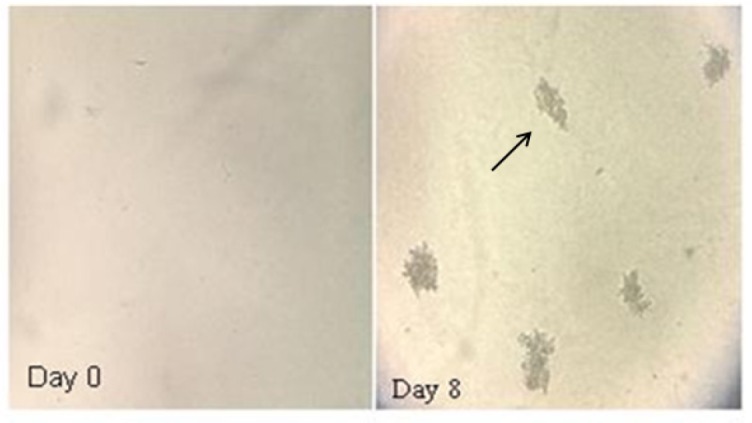
Representative Images of Colonies Formed in Soft Agar by Pari-ICR at day 0 and day 8. Single cell suspensions from 100 to 1000 cells per well were prepared. After 10 days, the number of colonies containing more than 50 cells was counted by an inverted microscope

To evaluate the ability of the established cell line to grow into a colony, the colony formation assay was used. After 3 weeks, Pari-ICR formed large colonies on agar with mean efficiency of 30% (Figure 4).

**Figure 5:**
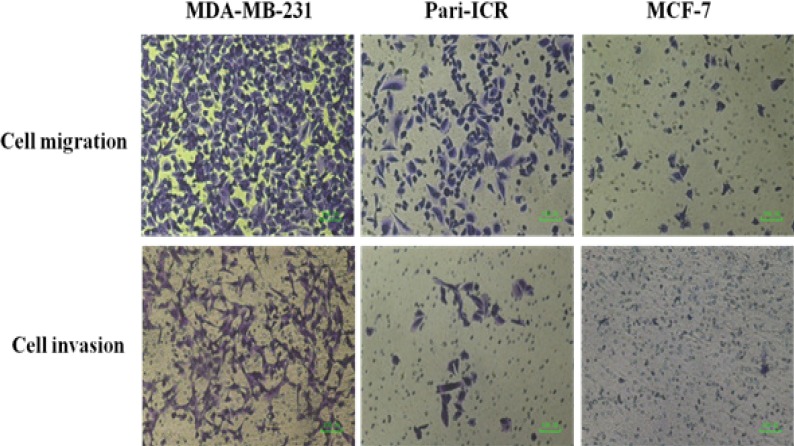
Migration and Invasion Assays of Pari-ICR Compared to MDA-MB-231 (Positive Control) and MCF-7 (Negative Control) Cell Lines

**Figure 6:**
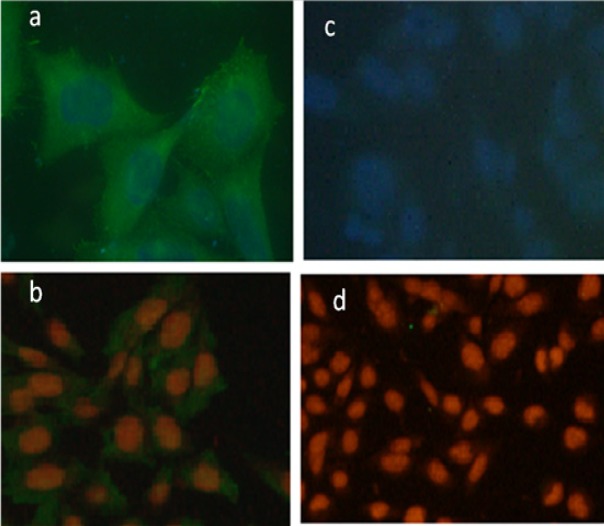
Immunofluorescence Staining of Pari-ICR Cell Line. Expression of Ezrin and Vimentin in pari-ICR was investigated by imunofluorescence assay. (a) Immunofluorescence staining with anti-Ezrin antibody, (b) Immunofluorescence staining with anti-Vimentin antibody, (c,d) Negative controls. Nucleus is stained by DAPI in figures (a) & (c), and by PE in figures (b) & (d)

**Figure 7:**
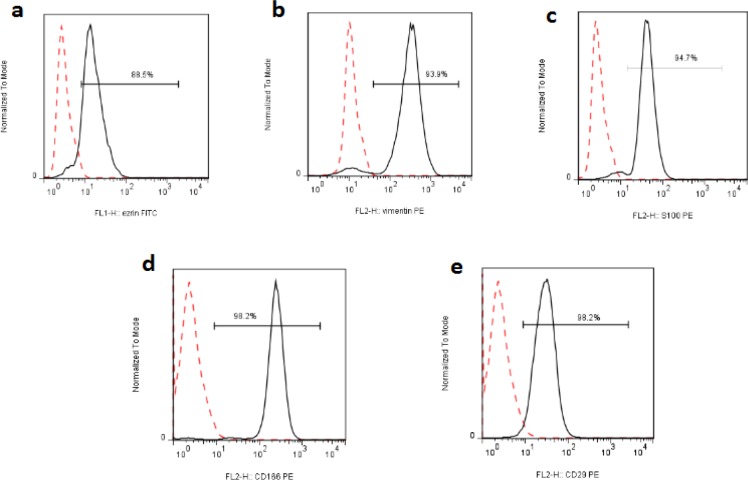
Expression of Different Markers by FACS in the Pari-ICR Cell Line. Pari-ICR cells were incubated with indicated antibodies, and analyzed by flow cytometery. a) Ezrin, b) Vimentin, c) S100, d) CD166, e) CD29. Dashed lines represent isotype control staining

**Figure 8:**
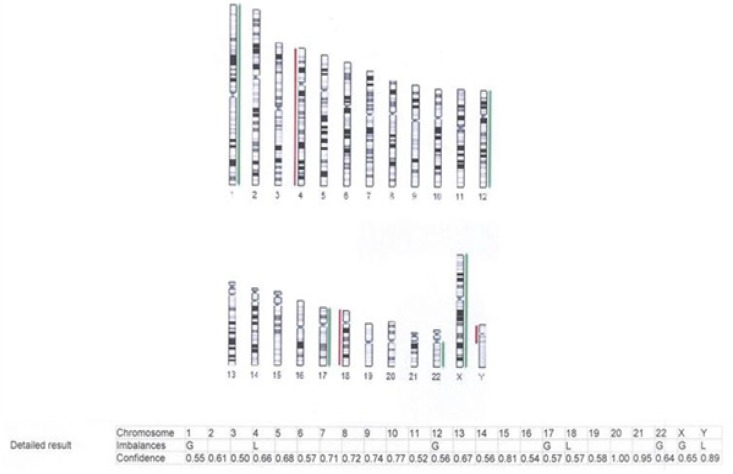
Array CGH of Pari-ICR Cell Line Shows Loss on Chromosomes 4, 18, Y and Gain on Chromosomes 1, 12, 17 and X

*Migration and invasion assay*

Cell migration and invasion assay was performed in order to investigate this ability of Pari- ICR cell line in comparison to MDA-MB231 and MCF-7 breast cancer cell lines. As shown in Figure 5, the migrated cell numbers of Pari-ICR cell line was higher than MCF-7 but lower than MDA-MB231.

*Phenotypic profiling of established cell line*

By immunofluorescent staining we observed that Pari-ICR cell line expressed a high level of Vimentin and Ezrin but not Cytokeratin19, Pan-cytokeratin, Nestin, and Desmin (Figure 6).

Flowcytometric analysis showed that Pari-ICR cell line was positive for Vimentin, Ezrin, S100, CD166, and CD29 but negative for EPCAM, Pan-cytokeratin, CD24, CD44, CD10, CD45, CD34, CD105, CD133, and CD146 (Figure 7).

*Chromosomal analysis of the cell line*

To detect gain and loss of chromosomes, comparative genomic hybridization array was performed and showed chromosomal losses on chromosomes 4 and 18 and gains on chromosomes 1, 12, and17 (Figure 8).

## Discussion

Breast cancer is a public health problem worldwide. It is a very heterogeneous disease, which has different biological features and clinical implications that lead to heterogeneous responses to therapy (Dai et al., 2015). Although, new approaches have been recently presented for the diagnosis and treatment, breast cancer accounts for 22% of new cancer cases each year (Pires et al., 2016). Moreover, although 5-year survival rate of breast cancer in developed countries is 85%, it is around 50-60% in developing countries (Movahedi et al., 2012). 

*In vitro *and* in vivo* models are important for providing additional information in breast carcinogenesis mechanisms and testing therapeutic strategies. Among the various models, cancer cell lines are the most widely used models. The cell lines are a pure population of cells and an infinite source of material, which can be applied easily to provide reproducible results with the same protocol (Burdall et al., 2003; Van Staveren et al., 2009; Turin et al., 2014). The majority of cancer cell lines have been passaged thousands of times (Shen et al., 2009), the process that may change their characteristics. Therefore, newly established cancer cell lines derived from primary tumors are always in demand.

In this study, we established a new triple negative breast cancer cell line, Pari-ICR, derived from a primary tumor of a young patient with invasive ductal carcinoma. The newly established cells grew well as an adherent monolayer in DMEM/F12 medium without any extra essential components for growth. Pari-ICR has spindle-shaped morphology like MDA-MB-231. The doubling time of Pari-ICR was 22h that is faster than the proliferation rate of many other breast cancer cell lines (Engel and Young, 1978). The immunocytochemistry staining revealed that the established cell line was negative for ER, PR, and HER2. Pari-ICR was negative for epithelial markers such as cytokeratin 19, pan-cytokeratin and EpCAM but it was strongly positive for Vimentin. 

Keratins are normally expressed in all types of epithelial cells and their neoplastic derivatives. Vimentin is an intermediate filament that is expressed in a wide variety of mesenchymal cell types and has important effect during invasion and migration. Several studies have reported that Vimentin is overexpressed in a large number of cancers including breast cancer. It has been shown that Vimentin expression had a significant correlation with the loss of one or more cytokeratins in malignant cells (Sommers et al., 1994; Satelli and Li, 2011; Vassilopoulos et al., 2014). Thomas et al. reported that Vimentin immunopositivity in breast tissues was inversely associated with keratin expression and directly associated with poor differentiation, negative estrogen/progesterone receptor status, and poor prognosis (Thompson et al., 1992; Choi et al., 2016). Although histogenesis of the Vimentin expressing tumors is unclear, myoepithelial histogenesis has been proposed. Moreover, it has been suggested that Vimentin expression can be considered as a sign of epithelial-mesenchymal transition (EMT), reflecting the final step of tumor dedifferentiation, which is generally associated with a high potential of tumor cell invasion (Korsching et al., 2005; Satelli and Li, 2011)

Sommers et al., (1994) have shown that human breast cancer cell lines were distributed along a spectrum of differentiation from well differentiated (epithelioid cell lines) to poorly differentiated (fibroblastic cell lines). They showed that fibroblastic cell lines such as MDA-MB-231 expressed Vimentin whereas cell lines such as MCF-7 and SKBR3 did not. The cell lines that were positive for Vimentin had reduced levels of keratins. In addition, Vimentin expression was associated with in-vitro invasiveness. Accordingly, overexpression of Vimentin in Pari-ICR may indicate the high metastatic potential of this cell line.

Ezrin, CD166 and CD29 are other markers that were expressed by Pari-ICR. Ezrin is a member of ERM family (Ezrin, Radixin, Moesin) that links the plasma membrane to the cytoskeleton of the cell. Erzin is detectable in normal and malignant cells and is involved in several functions such as cell migration, proliferation, survival/apoptosis, and adhesion (Meng et al., 2010; Gamei et al., 2014). Overexpression of this protein correlates with metastasis and poor prognosis in various types of human cancers including breast cancer (Ghaffari et al., 2014). A recent study demonstrated that inhibition of Ezrin with Ezrin shRNA in MCF-7 cell line decreased cell motility and invasiveness (Li et al., 2010).

Activated leukocyte cell adhesion molecule (ALCAM) also known as CD166 is a glycoprotein belonging to the immunoglobulin superfamily of adhesion molecules. CD166 functions as a cell-cell adhesion molecule in hemophilic (ALCAM-ALCAM) and heterophilic (ALCAM-CD6) interactions (Davies and Jiang, 2010). This glycoprotein, which is expressed in a wide variety of cell types, often shows increased expression in certain cancers including melanoma, colorectal, prostate, breast, ovarian, bladder, and esophageal cancer suggesting that CD166 is under restricting regulation during carcinogenesis (Davies and Jiang, 2010; Levin et al., 2010; Xiao et al., 2017). On the other hand, it has been shown that inhibition of CD166 promotes migration and invasion of tumor cells (Rosso et al., 2007; Kijima et al., 2012; Fujiwara et al., 2014). In breast cancer, the findings are controversial. While expression of ALCAM protects breast cancer cells against programmed cell death, its overexpression reduces the growth and migration of the tumor cells (Thompson et al., 1992; Jezierska et al., 2006; Rosso et al., 2007; Davies and Jiang, 2010). CD29, also known as Integrin β1, is a protein involved in cell motility and cancer metastasis. The malignant breast cancer cell line, MDA-MB-231, expresses high levels of β1 integrins, which promote invasive properties, whereas non-invasive MCF-7 cell line expresses low levels of β1 integrins (Hermann et al., 2016). It has been shown that the lack of CD29 results in a decreased cell migration (Vassilopoulos et al., 2014). Migration/invasion assays revealed that the established cell line had migration and invasiveness ability that was higher than MCF-7 but lower than MDA-MB231. This property of Pari-ICR cell line was in accordance with the expression of CD29.

Another marker expressed by Pari-ICR was S100. The S100 gene family, which is comprised of over 20 members, encodes low molecular weight calcium-binding proteins (Choi et al., 2016). They participate in a wide range of biological processes such as proliferation, migration and/or invasion, inflammation, and differentiation (Bresnick et al., 2015). Recent data suggest that specific S100 family members are engaged in cancer progression, especially metastasis (McKiernan et al., 2011). Recent studies indicated that abnormal expression of S100 proteins – mainly including S100A2, S100A4, and S100A7 – is related to metastasis of breast tumor(McKiernan et al., 2011; Li et al., 2014). The anchorage-independent growth in semisolid media is a property of most transformed cells that is correlated with tumorigenicity and metastatic potential. The established cell line was able to grow as colonies in the soft agar.

In conclusion, an immortalized triple negative breast cancer cell line was established from a primary breast tumor that exhibited high growth potential. Compared to other breast cancer cell lines that have been derived from a metastatic tumor, Pari-ICR seems to be a more plausible model. This breast cancer cell line can be served as a model for understanding molecular mechanisms of breast carcinogenesis in young patients. Moreover, it can be used as an appropriate source to find novel biomarkers and for assessment of new drugs.

## Statement conflict of Interest

The authors have no conflict of interest to declare.
